# African midwifery students’ self-assessed confidence in antenatal care: a multi-country study

**DOI:** 10.1080/16549716.2019.1689721

**Published:** 2019-11-21

**Authors:** Ingegerd Hildingsson, Helena Lindgren, Annika Karlström, Kyllike Christensson, Lena Bäck, Christina Mudokwenyu–Rawdon, Margaret C. Maimbolwa, Rose Mjawa Laisser, Grace Omoni, Angela Chimwaza, Enid Mwebaza, Jonah Kiruja, Bharati Sharma

**Affiliations:** a Department of Women’s and Children’s Health, Uppsala University, Uppsala, Sweden; b Department of Nursing, Mid Sweden University, Sundsvall, Sweden; c Department of Women’s and Children’s health, Karolinska Institutet, Stockholm, Sweden; d White Ribbon Alliance Zimbabwe, Harare, Zimbabwe; e School of Nursing Science, University of Zambia, Lusaka, Zambia; f Archbishop Antony Malaya School of Nursing, Catholic University of Health and Allied Science, Mwanza, Tanzania; g School of Nursing Science, University of Nairobi, Nairobi, Kenya; h Kamuzu College of Nursing, University of Malawi, Lilongwe, Malawi; i LAMRN, Kampala, Uganda; j College of Health Science and Medicine, Faculty of Nursing and Midwifery, Hargeisa University, Hargeisa, Somaliland; k Department of Nursing, Dalarna University, Falun, Sweden; l Indian Institute of Public Health, Gandhinagar, India

**Keywords:** Midwifery students, confidence, education

## Abstract

**Background**: Evidence-based antenatal care is one cornerstone in Safe Motherhood and educated and confident midwives remain to be optimal caregivers in Africa. Confidence in antenatal midwifery skills is important and could differ depending on the provision of education among the training institutions across Africa.

**Objective**: The aim of the study was to describe and compare midwifery students’ confidence in basic antenatal skills, in relation to age, sex, program type and level of program.

**Methods**: A survey in seven sub-Saharan African countries was conducted. Enrolled midwifery students from selected midwifery institutions in each country presented selfreported data on confidence to provide antenatal care. Data were collected using a selfadministered questionnaire. The questionnaire consisted of 22 antenatal skills based on the competency framework from the International Confederation of Midwives. The skills were grouped into three domains; Identify fetal and maternal risk factors and educate parents; Manage and document emergent complications and Physical assessment and nutrition.

**Results**: In total, 1407 midwifery students from seven Sub-Saharan countries responded. Almost one third (25-32%) of the students reported high levels of confidence in all three domains. Direct entry programs were associated with higher levels of confidence in all three domains, compared to post-nursing and double degree programs. Students enrolled at education with diploma level presented with high levels of confidence in two out of three domains.

**Conclusions**: A significant proportion of student midwives rated themselves low on confidence to provide ANC. Midwifery students enrolled in direct entry programs reported higher levels of confidence in all domains. It is important that local governments develop education standards, based on recommendations from the International Confederation of midwives. Further research is needed for the evaluation of actual competence.

## Background

Since the Safe Motherhood initiative in 1987 [1] several proposals, procedures, and guidelines have been developed to guarantee that pregnant women and new mothers receive sufficient and evidence-based antenatal, intrapartum and postpartum care [2]. This would result in optimal health for the woman and her unborn baby during pregnancy. A global access to midwifery care could prevent several maternal and new-born deaths [3]. Currently, WHO recommend that pregnant women should be offered a minimum of eight antenatal visits, in order to reduce perinatal mortality and also to improve women’s experiences of care [4]. The content of antenatal care (ANC) should include health check-ups, counselling about diet and physical activity, prevention of neonatal mortality, information about iron and folic acid supplement, tetanus vaccination and one early ultrasound examination [5]. There is a call for skilled health professionals worldwide, and educated midwives are the most appropriate caregivers for women during pregnancy and childbirth [6,7].

Shortage of qualified health professionals in Africa is a known cry, which could negatively impact the health of women and their new-born babies. For instance, maternal mortality is still high in low resource countries [8]. The shortage of qualified midwives attracts inadequate care from uneducated health-care providers who normally cover the gap, as the health facilities could be far away from women’s homes [9]. The Lancet series of midwifery concludes that ‘Midwifery and midwives are crucial to the achievement of national and international goals and targets in reproductive, maternal, new-born, and child health; now and beyond 2015’ [7], but this conclusion may not be realised by many African countries due to limited countries resource constraints for training and deployment.

In order to become a skilled and confident midwife, the provision of education is of utmost importance. The International Confederation of Midwives (ICM), in collaboration with WHO provides standards for midwifery education [6,10]. According to ICM, there are two educational pathways that leads to a midwife according to ICM standards [6], a direct-entry education (where no nursing education is required), and a post-nursing education. There are, however, some countries that use an integrated degree of nurse-midwifery education (double degree). The average length of a direct entry program is 3 years and a post-nursing education usually covers 18 months to 2 years. The double degree is usually 3–4 years long. ICM recommends a minimum of 3 years’ education within a direct entry education, or at least 18-months post-nursing midwifery education program [10]. Midwifery education leading to an integrated double degree in nursing and midwifery is common in India and other parts of Southeast Asia as well as in many African countries [9]. The scope of practice of midwifery, as defined by ICM, has resulted in recommendations of basic skills that any midwife should be able to know and to perform [11].

The concept of Confidence could mean that a person is certain about managing, e.g. work, family, social events, or relationships [12]. Some aspects of confidence are related to competence but are not synonymous [13]. Fullerton and co-workers describe confidence as an achievement, e.g. “an ability to do something successfully or effectively“ [14]. Confidence has some attributional factors: it is situation-based, which means that it is dependent on time and recourses. When related to education confidence could be related to the pedagogical level and structure, and confidence also relates to personal characteristics, attitudes, and motivation [14–18]. A high level of confidence is not directly proportional to high competence, but a low degree of confidence can be reflected in how skilled actions are performed [16].

Antenatal care is critical in attainment of improved maternal health outcomes and educated and confident midwives are the optimal caregivers [7,8]. However, there are limited data is available about the confidence of midwifery students in provision of ANC in Africa. Confidence in midwifery skills could differ depending on the organisation, length, and level of education. Confidence could also differ in different areas of midwifery care and how many students are exposed to the clinical areas of midwifery. Therefore, the aim of this study was to describe and compare midwifery students’ confidence in basic antenatal skills, in relation to age, sex, program type and level of program.

## Methods

### Design

A multi-country cross-sectional study of final year midwifery students, shortly before graduation.

### Setting

Midwifery programs from a variety of Sub-Saharan countries (Kenya, Zambia, Uganda, Tanzania, Somaliland, Malawi and Zimbabwe). These countries are part of the Lugina African Midwives Research Network (LAMRN) (www.lamrn.org). The institutions holding the midwifery programs could be governmentally funded, private or public.

### Participants

Midwifery students in the final semester of their education were asked to participate. To be included in the study, the students were enrolled as direct entry, post-nursing or integrated nurse-midwifery education at the minimum of diploma level.

### Data collection

Data were collected using a self-administered questionnaire which was adopted from an Indian study conducted by Sharma et al. in 2014 [9]. The questionnaire was reviewed and revised in a workshop for methods and tool development where all the principle investigators from each country participated. Only minor corrections in the phrasing of background data were performed, to fit each country context.

The original questionnaire by Sharma et al. [9], consisted of four competency areas from the International Confederation of Midwives in 2013; Antenatal care (22 skills), Intrapartum care (46 skills), Newborn care (19 skills) and Postpartum care (16 skills). For this paper, only the 22 questions about confidence in antenatal care (ANC) were investigated together with some background data. The background data included students’ age, sex, level of program (bachelor or diploma), and type of program (direct entry, post-nursing, and integrated nurse-midwifery education).

The students assessed confidence for each skill statement on a four-point scale. The question on confidence read as ‘How confident are you to perform this skill independently’? 1 = Not confident, 2 = Somewhat confident, 3 = Confident, 4 = Very confident.

### Process

The questionnaire was self-administered in a classroom situation with a researcher present. The researcher first introduced the questionnaire and was available for clarifications. The data were collected 2016–17 and matched the period when the students have completed their education and were about to complete training.

### Analysis

The Statistical package of Social Science (SPSS) version 24 (SPSS, Inc., Chicago, (USA)) was used to analyze and managing the data. Descriptive statistics were used to present data. A principal component analysis (PCA) with oblimin rotation was performed for identifying domains that could reduce the number of statements [19]. The factorability of the data was assessed through the Kaiser Meyer Olkin measure of sampling adequacy (KMO) which should be over 0.6, and Bartlett’s test of sphericity to be statistically significant. In this dataset, the KMO value was .95 and Bartlett’s test of sphericity reached statistical significance (<0.000), which supported the sample factorability and adequacy. Then, the number of retained domains was guided by the Kaisers criterion (eigenvalues >1), and also Cattell’s scree test and inspection of the scree plot. All the components with an eigenvalue 1 and statements loading above 0.40 were included. Finally, Cronbach alpha coefficients were calculated for each of the domains, to assess reliability. The principal component analysis resulted in three domains of antenatal care that were labelled; *Identify fetal and maternal risk factors and educate parents; Manage and document emergent complications* and *Physical assessment and nutrition*.

When the domains were identified, a one-way analysis of variance (ANOVA) was performed to assess the impact of background variables on the variation in the domains. Thereafter, the domains were dichotomized into two groups and students who scored above the 75^th^ percentile were compared with students who scored lower. Odds ratios with a 95% confidence interval were calculated for each of the dichotomized domains for the explanatory background variables. In order to investigate which factors contributed most strongly to be very confident (75^th^ perc), all variables were entered in the logistic regression model for each of the dichotomized domains and removed one by one until only statistically significant variables remained.

## Results

In total, 1407 midwifery students from seven Sub-Saharan countries were included. The majority were between 26 and 35 years old and female. The majority of students were enrolled in a post-nursing program, 27% in a direct entry program, and 29% in an integrated nurse-midwifery program. The length of the education ranged from 12 to 48 months and the majority of education was on a diploma level (Table 1). Three countries had only one type of education (integrated, direct entry and post nursing, respectively), two countries offered post nursing and integrated nurse-midwifery education and two countries post nursing and direct entry.

**Table 1.** Study participants.

**Age groups**	
18–25 years	638 (45.7)
26–35 years	590 (42.2)
36–60 years	167 (11.9)
**Sex**	
Males	237 (16.9)
Females	1165 (83.1)
**Type of midwifery program**	
Direct entry	376 (26.8)
Post-nursing	607 (43.3)
Integrated nurse-midwifery	420 (29.9)
**Level of program**	
Bachelor	449 (32.0)
Diploma	954 (68.0)
**Lenght of education**	
12 months	416 (29.7)
18 months	192 (13.7)
24 months	245 (17.5)
36 months	156 (11.1)
42 months	117 (8.3)
48 months	277 (19.7)

In Table 2 the results from the analysis of variance of the three domains are presented. There were statistically significant differences between the age groups and the three domains, with students aged 26–35 years reporting the highest levels of confidence in all three domains. Female students were more confident than males for all three domains. Students allocated at a direct entry program scored higher on all the domains. Students enrolled at a program on a bachelor level were less likely to be confident compared to students in diploma programs in the domain *Identify fetal and maternal risk factors and educate parents* and *Physical assessment and nutrition*. No difference was found in the domain *Manage and document emergent complications*.

**Table 2.** Mean scores (SD) of the domains of antenatal care in relation to background variables.

	Identify fetal and maternal risk	Manage and document	Physical assessment
	factors and educate parents	emergent complications	and nutrition
**Age groups**			
18–25 years (n = 638)	38.92 (5.03)	9.1 (2.44)	31.04 (4.08)
26–35 years (n = 590)	38.96 (4.79)	10.02 (1.88)	32.04 (3.85)
36–60 years (n = 167)	37.58 (5.46)	9.72 (2.19)	30.81 (4.46)
p-value	0.004	<0.001	0.002
**Sex**			
Males (n = 237)	38.02 (5.29)	8.98 (2.37)	31.32 (4.25)
Females (n = 1165)	38.94 (4.92)	9.72 (2.13)	31.96 (4.01)
p-value	0.010	<0.001	0.011
**Type of midwifery program**			
Direct entry	39.42 (4.80)	10.12 (2.00)	32.36 (3.95)
Post-nursing	38.78 (4.90)	10.06 (1.79)	31.91 (3.84)
Integrated nurse-midwifery	38.25 (5.25)	8.45 (2.44)	31.27 (4.39)
p-value	0.004	<0.001	0.001
**Level of program**			
Bachelor level	38.35 (5.47)	9.64 (2.15)	31.33 (4.57)
Diploma level	38.99 (4.75)	9.57 (2.21)	32.07 (3.82)
p-value	0.026	0.568	0.001
Cronbach alpha	0.90	0.77	0.87

About 25–32% of the students reported high levels of confidence (>75^th^ perc) for the three domains (Table 3). Age was not commonly associated with high confidence, with the exception that older students were less likely to be confident in the domain *Identify fetal and maternal risk factors and educate parents*. Female students reported higher confidence compared to males in *Manage and document emergent complications*.

**Table 3.** Confidence in basic midwifery skills in relation to background.

	Identify fetal and maternal risk factors and educate parents			Manage and document emergent complications			Physical assessment and nutrition		
	Higher	Lower		Higher	Lower		Higher	Lower	
	confidence	confidence	Odds Ratio	confidence	confidence	Odds Ratio	confidence	confidence	Odds Ratio
	n (%)	n (%)	(95% CI)	n (%)	n (%)	(95% CI)	n (%)	n (%)	(95% CI)
**Age groups**									
18–25 years (n = 638)	206 (46.9)	432 (45.4)	0.94 (0.74–1.20)	181 (43.9)	457 (46.5)	0.83 (0.63–1.07)	169 (48.0)	469 (45.1)	1.04 (0.80–1.34)
26–35 years (n = 590)	197 (44.5)	302 (41.2)	1.0 Ref.	189 (45.9)	400 (40.7)	1.0 Ref.	151 (42.9)	437 (42.9)	1.0 Ref.
36–60 years (n = 167)	40 (9.0)	127 (13.4)	0.62 (0.42–0.92)*	42 (10.2)	125 (12.7)	0.71 (0.48–1.05)	32 (9.1)	135 (13.0)	0.68 (0.48–1.05)
**Sex**									
Males (n = 237)	64 (14.3)	173 (18.1)	1.0 Ref.	52 (12.6)	185 (18.7)	1.0 Ref.	49 (13.9)	188 (18.0)	1.0 Ref.
Females (n = 1165)	382 (85.7)	782 (81.9)	1.32 (0.96–1.80)	362 (87.4)	802 (81.3)	1.60 (1.15–2.23)**	304 (86.1)	859 (82.0)	1.35 (0.96–1.80)
**Type of midwifery program**									
Direct entry (n = 376)	146 (32.7)	229 (24.3)	1.42 (1.08–1.86)*	154 (37.1)	221 (22.3)	1.61 (1.23–2.11)*	128 (36.2)	246 (23.5)	1.70 (1.25–2.26)***
Post-nursing (n = 607)	188 (44.5)	419 (43.6)	1.0 Ref.	183 (44.1)	424 (43.0)	1.0 Ref.	142 (40.1)	465 (44.4)	1.0 Ref.
Integrated nurse-midwifery (n = 420)	113 (25.3)	307 (32.1)	0.82 (0.62–1.05)	78 (18.8)	34 (34.7)	0.52 (0.39–0.71)***	84 (23.7)	336 (37.1)	0.81 (0.60–1.10)
**Level of program**									
Diploma (n = 954)	318 (71.1)	636 (66.6)	1.23 (0.96–1.57)	291 (70.1)	663 (67.2)	1.14 (0.84–1.47)	268 (75.7)	695 (65.4)	1.54 (1.25–2.16)***
Bachelor (n = 449)	129 (28.9)	319 (33.4)	1.0 Ref.	124 (29.9)	324 (32.8)	1.0 Ref.	86 (24.3)	362 (34.6)	1.0 Ref.

* = p < 0.05, ** = p < 0.01, *** = p < 0.001.

There were considerable differences between type of program and level of education (Table 3). Direct entry programs were associated with higher levels of confidence in *all* domains.

Students enrolled at education with diploma level presented with high levels of confidence in the domain *Physical assessment and Nutrition*.

In order to investigate which factors contributed most strongly to be very confident, the final regression model showed that direct entry was the variable that contributed most in confidence for all three dichotomized domains (Table 4). Being at older age, integrated nurse-midwifery education and diploma level decreased the chance of being very confident in some of the domains.

**Table 4.** The most important factors for being very confident in antenatal care.

	Identify fetal and maternal risk factors and educate parents	Manage and document emergent complications	Physcial assessment and nutrition
Age >35 years	0.6 (0.4–0.9)*		
Direct entry prog	1.4 (1.2–1.8)*	1.8 (1.3–2.3)***	1.70 (1.3–2.3)***
Integrated nurse-midwifery		0.4 (0.3–0.5)***	
Diploma level		0.5 (0.4–0.8)***	

* = p < 0.05, *** = p < 0.001

## Discussion

The main findings indicated that the type and level of midwifery program were associated with students’ self-assessed confidence in some basic skills in the area of antenatal care.

The results favoured direct entry programs and diploma level. Background variables showed that female students and those aged 26–35 years were more confident compared to their male counterparts and students younger or older.

### High levels of confidence

The results showed that one third to one fourth of the students in the present study scored high on confidence in the three domains. This is quite similar to the findings from India, where Sharma and co-authors [13] found that 23–28% scored above the 75^th^ percentile.

### Program type

Based on the results from the present study it became obvious that direct entry programs were successful in contributing to the fact that midwifery students perceived high confidence in all areas of antenatal care. This might be understood from the strong focus on maternal, child and family health and not on diseases such as in nursing. Similar findings have been reported in an Italian study where a direct entry midwifery education has been offered since the mid 1990s [20]. The results showed that students without a prior education focused more on the integration and pathophysiology of the basic care for maternal and child health and learned the skills integrated for their future scope of practice. However, the result in the Italian study also showed that the academic curriculum with limitations in hours allocated to the program impacted negatively on the learning process in terms of limited time for reflection which resulted in students postponing their graduation [20].

The integrated nurse-midwifery education was associated with lower confidence in how to manage emergent complications during pregnancy. Confidence in this area might not only be associated with program type, but also with the clinics with students’ practice facilities, the referral systems and transports as mentioned by in an Indian grounded theory study [21]. In the Indian study, midwives educated within the system of integrated nurse-midwifery (not acknowledged by the ICM) reported that the clinical education was marginalized and the students only got exposed to the restricted midwifery practice of staff nurses.

### Program level

Students in the diploma programs reported higher levels of confidence in two of the three domains. These findings are similar to those reported from the Indian study [22], where diploma students were 2–4 times more likely to have high confidence, compared to students on bachelor level. It is not surprising that the transition of midwifery from being an overall clinical into an academic profession with high requirements for clinical skills has an impact on students’ self-concept. Hylton and co-authors [23] have investigated the experience of nurse students after the introduction of a bachelor degree in nursing and found that there is a conflict between the concrete and practical knowledge and the ability to grasp conceptual knowledge.

The skills that are expected from nurses and midwives by colleagues in the clinical area might mostly be related to the ability to manage the daily work at the labour ward. Nevertheless, Renfrew et al. [3] have underlined the importance of evidence for the effectiveness of midwifery care practice in the Lancet series of Midwifery. Higher education is a pathway for midwives to leadership roles, which is of importance for the development of reproductive health care in a long-term perspective [24]. On the other hand, the students on bachelor level reported a higher level of confidence in emergency situations in this study. Nieuwenhuijze et al. [25] argue that academic development is required in order to understand the complexity of situations in maternity care. It could be assumed that students with a bachelor degree achieve a higher awareness of such complexity and that the finding mirrors their own sense of capability taking the lead in difficult situations. Another explanation could be a more critical self-appraisal trained during the university-based education.

### Age and sex

In the present study, we found that the most confident students were aged 26–35 years. Few studies have focused on age and confidence in midwifery skills. There could also be a relation between type of program and age, as direct entry programs more often attract younger students and post-nursing students are older by nature. According to young age, an Australian study interviewed young midwifery students (<21 years) at a direct entry education. The researchers found most advantages in being young, despite the emotional demanding education and profession. The result of that study showed that young midwifery students developed resilience and coping strategies over time, they also demonstrated commitment to a career in midwifery [26]. Similarly, younger Swedish midwifery students (all post-nursing programs) were more confident in the majority of skills investigated, compared to older students [27].

Female students in the current study were more likely to be confident in the antenatal skills compared to male students. Research on the impact of gender are somewhat inconclusive. An integrated review showed that gender did not have any impact on quality of pre-service education of health providers [28], while an Ethiopian study [29] found that male midwifery students scored higher in, e.g. antenatal care, compared to females using OSCE (Objective Structured Clinical Examination).

This study has several limitations. First, it is compromised by its observational design, which makes it difficult to draw any final conclusions about cause and effect. Also, we do not know how many students were present in the classroom when they filled out the questionnaire. It is likely that a high percentage were available at the time of data collection as this coincided with their concluding weeks of education where they had their final examinations. Another limitation was that the confidence of the skills was self-assessed and there is no way to control the actual competence. The presence of a researcher during data collection could have resulted in social desirable answers, but also gave the students the opportunity to ask questions. The extensive questionnaire was time-consuming, but with few incomplete answers. The dichotomization of the continuous variables based on the 75^th^ percentile could be questioned, as it is not based on any standard, only on the participants’ scores. One difficultness was the harmonization of background variables. Although using the same questionnaire, the countries were free to adjust the background variables to fit the local circumstances (e.g. the number of births attended, classroom versus hands-on training). It is therefor important that all midwifery educations globally follow the recommendations from ICM.

Nevertheless, the multi-country approach and the large number of respondents make the data reliable. The questionnaire has been used in other studies [13,22,27], and further developed and refined by a group of senior researchers for the current study. This strengthen the validity of the tool.

## Conclusion

The result showed an association between high levels of confidence in antenatal care, type of program and program level of the education. Midwifery students enrolled in direct entry programs reported higher levels of confidence in all domains. Further research is needed for evaluation of application of actual competences to clients. This is the first paper about confidence in African midwifery students. Papers about students’ confidence in intrapartum and postpartum care will follow. All these papers will add to the growing body of studies about midwifery education and could form a basis for discussion with policy makers in each country.

## Clinical implications

In summary, there are large weaknesses in the organization of midwifery education globally. It is important that local governments develop education standards, based on recommendations from the ICM (e.g. number of births attended, proportion of classroom teaching and hands-on training). Compared to other health educations, midwifery education has been treated differently with previous attempts to solve the lack of trained midwives by replacing them with ‘short and dirty’ educations that did not favour maternal and newborn health.

## Paper context

In low resource settings with high maternal and infant mortality, midwives are the key and recommended health providers for antenatal care. Confident midwives are essential for providing high quality care. This paper highlights the importance of direct entry program and diploma level as the basis for students’ perception of confidence in midwifery skills. Local governments should develop education standards, based on recommendations from the International Confederation of Midwives.
